# Visual processing and decision-making in autism and dyslexia: Insights from cross-syndrome approaches

**DOI:** 10.1177/17470218241264627

**Published:** 2024-07-27

**Authors:** Catherine Manning

**Affiliations:** 1 School of Psychology and Clinical Language Sciences, University of Reading, Reading, UK; 2 School of Psychology, University of Birmingham, Birmingham, UK

**Keywords:** Motion perception, decision-making, cognitive modelling, diffusion model, equivalent noise, developmental conditions

## Abstract

Atypical visual processing has been reported in developmental conditions like autism and dyslexia, and some accounts propose a causal role for visual processing in the development of these conditions. However, few studies make direct comparisons between conditions, or use sufficiently sensitive methods, meaning that it is hard to say whether atypical visual processing tells us anything specific about these conditions, or whether it reflects a more general marker of atypical development. Here I review findings from two computational modelling approaches (equivalent noise and diffusion modelling) and related electroencephalography (EEG) indices which we have applied to data from autistic, dyslexic and typically developing children to reveal how the component processes involved in visual processing and decision-making are altered in autism and dyslexia. The results identify both areas of convergence and divergence in autistic and dyslexic children’s visual processing and decision-making, with implications for influential theoretical accounts such as weak central coherence, increased internal noise, and dorsal-stream vulnerability. In both sets of studies, we also see considerable variability across children in all three groups. To better understand this variability, and further understand the convergence and divergence identified between conditions, future studies would benefit from studying how the component processes reviewed here relate to transdiagnostic dimensions, which will also give insights into individual differences in visual processing and decision-making more generally.

## Introduction to visual processing in autism and dyslexia

Autism and dyslexia are two developmental conditions which, on the surface, are quite distinct: autism is a condition most known for its effects on social communication and interaction, alongside “non-social” characteristics such as repetitive behaviours and specialised interests (American Psychiatric Association, 2013), whereas dyslexia is characterised by difficulties learning to read and spell (British Dyslexia Association, n.d.; Rose, 2009). Despite these distinct phenotypes, sensory processing, and more specifically visual processing—the focus of this review—has been linked to both conditions.

Sensory processing differences are recognised in the diagnostic criteria for autism (American Psychiatric Association, 2013) and are linked to everyday functioning and mental health (Ashburner et al., 2008; Carpenter et al., 2019; MacLennan et al., 2021; Rossow et al., 2021). While the diagnostic criteria refer to increased and/or reduced reactivity to sensory information and seeking out sensory stimulation across *all* sensory modalities, there is also an established body of work showing differences in visual perception between autistic participants and non-autistic participants (see Hadad & Yashar, 2022; Robertson & Baron-Cohen, 2017; Simmons et al., 2009, for reviews). For example, in autistic individuals there are reports of reduced sensitivity and recognition for faces (Griffin et al., 2021; Sasson, 2006), reduced sensitivity to complex motion stimuli (Van der Hallen et al., 2019), faster visual search (Constable et al., 2020; Kaldy et al., 2016) and a generally more detail-focused perceptual style (see Robertson & Baron-Cohen, 2017, for review). While a consensus has not yet been reached, some scholars propose that sensory differences, including in visual perception, may reflect neurobiological differences that are causal to the development of autism (Robertson & Baron-Cohen, 2017).

In contrast to autism, sensory processing differences are not part of the diagnostic criteria for dyslexia. However, differences in visual perception have been long-studied in this condition, with early accounts of dyslexia ascribing a causal role to visual processing difficulties (e.g., Morgan, 1896, see Kirby et al., 2020 for review) and a more modern account suggesting that differences in the development of the magnocellular system lead to the reading difficulties experienced by those with dyslexia (Stein, 2019; Stein & Walsh, 1997). While the causality of visual processing differences is debated (Kristjánsson & Sigurdardottir, 2023), there is clear evidence for visual perceptual differences in dyslexia, including reduced sensitivity to motion (Benassi et al., 2010), reduced sensitivity to flicker (Gibson et al., 2006) and atypical visuospatial attention (Bosse et al., 2007; Franceschini et al., 2012).

The study of visual perception in these developmental conditions normally uses the case-control approach, where performance of individuals with a single condition (e.g., autism *or* dyslexia) is contrasted with that of neurotypical participants, without comparing performance across multiple developmental conditions (although there are exceptions, for example, Pellicano & Gibson, 2008; Tsermentseli et al., 2008). Yet cross-syndrome approaches are relevant for elucidating the causal relationships between visual processing and the development of conditions, and understanding why conditions might overlap. For example, if visual processing is affected similarly in all developmental conditions, this might suggest that visual processing is a consequence of a brain that is developing differently rather than being causal to the development of the specific characteristics of each condition.

In this review, I will summarise evidence from two sets of studies which have presented the same paradigms to autistic, dyslexic, and typically developing children as a way of trying to better understand the development of component processes involved in visual processing and decision-making and whether these are affected in a similar or distinct way across autism and dyslexia. First, I will introduce the motion coherence paradigm, which has been extensively used in studies of visual processing in both autism (Van der Hallen et al., 2019, for meta-analysis) and dyslexia (Benassi et al., 2010, for meta-analysis), and explain why our understanding based on this paradigm is limited. Next, I will review findings from equivalent noise modelling, which aims to uncover the spatial limits to motion and orientation processing in autistic and dyslexic children. I will then present findings from diffusion modelling and electroencephalography (EEG) studies, which help better understand the temporal dynamics of processes leading to perceptual decisions. For each of these approaches, I will start with what we know from cross-sectional studies of typical development as a benchmark for understanding performance in autistic and dyslexic children. Finally, I will reflect on what these approaches together tell us about visual processing and decision-making in autism and dyslexia, and what future work is needed. This review focuses primarily on work in our group, as the first to apply these paradigms to both autistic and dyslexic children. However, I also review related work using similar paradigms, to provide a comprehensive overview.

## Motion coherence paradigm

The motion coherence task (Newsome & Paré, 1988) is a commonly used task to measure global motion processing ability in developmental populations. Global motion processing refers to the ability to combine motion signals over space and time to perceive the overall motion of elements which may each move in different directions, like a flock of birds. The motion coherence task requires participants to detect or discriminate the overall motion carried in a set of signal dots moving in a coherent direction amid randomly moving noise dots. This ability follows a protracted development across childhood (Gunn et al., 2002; Hadad et al., 2011), and has been shown to be affected in a range of conditions, including autism (Van der Hallen et al., 2019), dyslexia (Benassi et al., 2010), Williams syndrome (Atkinson et al., 1997, 2006), Fragile X syndrome (Kogan et al., 2004), and schizophrenia (Chen, 2011; Chen et al., 2003; Kéri & Kelemen, 2024). Individuals with these conditions have been reported, on average, to require a higher proportion of signal dots to perceive the overall motion, compared with neurotypical participants.

The fact that motion coherence thresholds are elevated in this range of conditions is consistent with the dorsal-stream vulnerability account (Atkinson, 2017; Braddick et al., 2003; Grinter et al., 2010). According to this account, the dorsal stream, which is important for performing global motion tasks, is particularly vulnerable to atypical development, leading to elevated motion coherence thresholds in a range of conditions. Meanwhile, form coherence thresholds, which more heavily tax the ventral stream, appear less affected (see Atkinson, 2017, for review). From this account, we might conclude that elevated motion coherence thresholds are a consequence of a brain developing differently, as opposed to reflecting a causal factor in the development of these conditions. However, there are various reasons why motion coherence thresholds could be elevated (Dakin & Frith, 2005), and these reasons could vary from one condition to the other. Therefore, in sections “Uncovering spatial parameters using equivalent noise modelling” and “Uncovering temporal dynamics using diffusion modelling and EEG,” I will introduce cross-syndrome modelling and EEG approaches that have helped uncover the underlying spatial and temporal parameters involved in global motion and orientation processing, and how these vary in autism and dyslexia.

## Uncovering spatial parameters using equivalent noise modelling

Although the motion coherence task is commonly termed a “global” motion task, performance in this task could also be limited by local processing, by which we mean how precisely the direction of each dot can be estimated (Dakin & Frith, 2005). Moreover, performance in motion coherence tasks could also be affected by a participant’s ability to filter out or ignore the randomly moving noise dots (“noise exclusion”; Dakin et al., 2005). The motion coherence task alone cannot distinguish between these possibilities.

**Figure 1.**
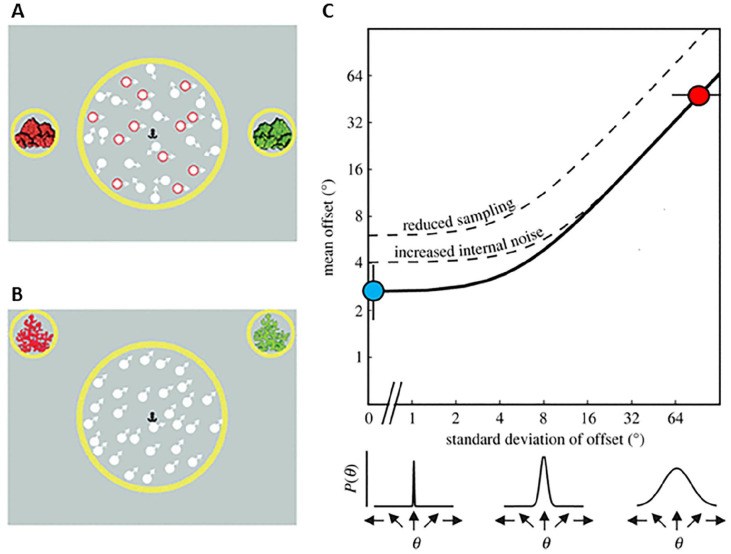
Schematic representation of motion tasks presented in studies using the equivalent noise approach (A–B) and the equivalent noise function (C). *Source*. Figure adapted from Manning, Hulks, et al. (2022). A. Schematic representation of a trial from the motion coherence task in which 40% of dots are signal dots moving in a coherent direction (rightward in this example, outlined in red for illustrative purposes) among randomly moving noise dots. The participant is asked to determine whether the overall motion is towards the red (left) or green (right) rocks. B. Schematic representation of a trial from the Gaussian motion task, in which the dot directions are taken from a Gaussian distribution with a standard deviation of 10° and mean offset of +45°. The participant is asked to determine whether the overall motion (i.e., mean offset) is towards the red (−45°) or green (+45°) reef. C. Example equivalent noise function relating direction discrimination thresholds to external noise (i.e., the standard deviation of dot directions presented in the Gaussian motion task (B)). Direction discrimination thresholds are relatively unaffected by low levels of external noise, as internal noise dominates. However, as external noise is increased further, the internal noise is swamped and thresholds start to increase. In our tasks with children, the equivalent noise function was constrained by data from two conditions. In the no-noise condition (blue), the standard deviation was fixed at 0° and the no-noise threshold was obtained by varying the mean offset. In the high-noise condition (red), the mean offset was fixed at ±45°, and the standard deviation was varied to find the maximum tolerable noise. Sampling and internal noise were then estimated. Reduced sampling shifts the function upwards, with reduced discrimination performance at all levels of internal noise. By contrast, increased levels of internal noise lead to higher thresholds at low levels of external noise and a rightwards shift of the elbow of the function, so that more external noise is required before thresholds start to increase.

The equivalent noise model allows us to quantify both local and global limits to motion perception, by estimating internal noise (i.e., the precision with which each dot direction is estimated) and sampling (i.e., how well motion signals across dots are averaged; Dakin et al., 2005). The logic behind equivalent noise analysis is that discrimination thresholds are limited by both internal noise and external noise (stimulus noise), and that internal noise levels can be estimated by investigating how discrimination thresholds vary as a function of external noise (Figure 1). The task used for this modelling differs from a motion coherence task: rather than having a separate distribution of signal dots and noise dots, the dot directions in a given trial are taken from a Gaussian distribution (see Figure 1). As in a motion coherence task, the participant is required to discriminate the overall direction of dots, but here the external (stimulus) noise—and hence the difficulty—is manipulated by varying the standard deviation of the Gaussian distribution from which the dots are taken. Importantly, this task also removes the need for noise exclusion, as there are no randomly moving dots that need to be filtered out—instead, the optimal strategy is to average across all dots. A summary of studies which have applied this paradigm to understand global motion processing in children is provided in Table 1.

**Table 1.** Summary of studies applying equivalent noise analysis to children’s global motion processing.

Study	Tasks	Sample	Key findings
Manning et al. (2014)	Motion coherence and Gaussian motion tasks for two speed conditions (1.5°/sec and 6°/sec)	Typically developing children aged 5 (*n* = 21), 7 (*n* = 27), 9 (*n* = 25) and 11 years (*n* = 20), and adults (*n* = 30)	• With age, internal noise decreases, sampling increases, and motion coherence thresholds decrease. • Internal noise is adult-like earlier than sampling. • Reduced motion coherence thresholds with age are driven by increases in sampling (not internal noise).
Bogfjellmo et al. (2014)	Gaussian motion task for two speed conditions (2.8°/sec and 9.8°/sec)	Children / young people aged 6 to 17 years (*n* = 103)	• Sampling increases with age, whereas internal noise does not significantly change. • Internal noise is adult-like earlier than sampling.
Manning et al. (2015)	Motion coherence and Gaussian motion tasks for two speed conditions (1.5°/sec and 6°/sec)	Autistic children (*n* = 33) and typically developing children (*n* = 33) aged 6-13 years	• Autistic children show accurate motion discrimination over a significantly greater range of external noise than typically developing children • Increased sampling estimates in autistic children, but no significant differences in internal noise or motion coherence thresholds. • No significant effect of speed condition or interaction with group.
Manning et al. (2017)	Motion coherence and Gaussian motion tasks with only two speed conditions (1.5°/sec). Also orientation coherence and Gaussian orientation tasks.	Autistic children (*n* = 46) and typically developing children (*n* = 45) aged 6-14 years	• Group difference in sampling for motion was not significant (cf. Manning et al., 2015), and inconclusive (BF = .35) • No evidence that the effect size for a group difference in sampling for motion differed from Manning et al. (2015) • Across Manning et al. (2015) and this study, evidence for increased sampling for motion (meta-analytic BF = 7.77) • No evidence for group differences in any parameters for orientation tasks
Manning, Hulks et al. (2022)	As in Manning et al. (2017)	Dyslexic children (*n* = 48) and typically developing children (*n* = 48) aged 8-14 years	• In motion tasks, dyslexic children had higher internal noise and higher motion coherence thresholds, but no group differences in sampling. • In orientation tasks, dyslexic children had higher orientation coherence thresholds but no significant differences in sampling and internal noise.

We first presented this task alongside a traditional motion coherence task to typically developing children aged 5-, 7-, 9-, and 11-years old and adults to understand how equivalent noise model parameters vary across age groups (Manning et al., 2014). While there was considerable between-participants variability for all age groups, we found that younger children, overall, had higher levels of internal noise and lower sampling estimates than older children and adults. Specifically, 5-year-olds had significantly higher internal noise than adults, whereas the older age groups did not. Sampling estimates were significantly lower in all child groups relative to adults when the stimuli were moving slowly (1.5°/sec), but only 5- and 7-year-olds had significantly lower sampling when stimuli were moving faster (6°/sec), showing a differential developmental trajectory for slow and fast stimuli. Importantly, it was specifically age-related differences in sampling ability that appeared to drive age-related increases in motion coherence thresholds, in both speed conditions. In the same year, using a similar approach but with stimuli moving at slightly faster speeds (2.8°/sec and 9.8°/sec), Bogfjellmo et al. (2014) reported increases in sampling in children aged 6 to 17 years, while internal noise levels stayed constant. These results complement our own findings, by suggesting that internal noise reduces to adult-like levels by around 6 or 7 years of age, while sampling may follow a more protracted development. Falkenberg et al. (2014) also reached a similar conclusion when applying equivalent noise analysis to children’s performance in a task requiring them to discriminate the direction of sinusoidal gratings.

We next sought to apply this model to understand the reasons for elevated motion coherence thresholds in autistic children (Manning et al., 2015). We had hypothesised that autistic children would show *reduced* sampling ability compared with typically developing children, in line with the Weak Central Coherence account of autism (Frith & Happé, 1994; Happé & Frith, 2006), alongside either increased (Simmons, 2019; Simmons et al., 2009) or reduced (Davis & Plaisted-Grant, 2015) levels of internal noise. However, surprisingly, we found that autistic children were able to accurately discriminate the overall motion direction over a *greater* range of external noise compared with age- and ability-matched typically developing children, consistent with *increased* sampling ability. Meanwhile, they showed no significant differences in internal noise, and no significant differences in motion coherence thresholds. As increased sampling leads to lower motion coherence thresholds in typical development (Manning et al., 2014), the fact that increased sampling in autistic children does not go hand-in-hand with reduced motion coherence thresholds suggests that autistic children may be limited in motion coherence tasks due to noise exclusion—a suggestion which has been supported by other research (Van de Cruys et al., 2017; Zaidel et al., 2015).

As these results were not as we had hypothesised, we conducted a replication study with a new set of autistic and typically developing children (Manning et al., 2017). We again found that autistic children had, overall, higher mean sampling estimates than typically developing children, but the effect size was smaller. The group difference was not significant in this replication dataset alone, and represented inconclusive evidence for either the null or alternative hypothesis (Bayes Factor [BF] = .35). However, there was also no evidence that the effect size in the replication sample differed from the original sample (inverse BF = 1.79), and when combining across both samples (*n* = 78 per group), we found strong evidence for increased sampling of motion information in autistic children (the meta-analytic BF reflected 7.77 times more evidence in support of the alternative hypothesis of group differences than the null hypothesis). In this replication study, we also presented corresponding orientation tasks, to determine if increased sampling ability extended to a static, form task that is typically associated with the ventral stream, following reports of increased (Dickinson et al., 2016) or comparable (Freyberg et al., 2016; Shafai et al., 2015) sensitivity to orientation in autistic relative to neurotypical individuals. Here, we found no evidence for group differences in any parameters, suggesting that increased integration does not extend to orientation processing in autistic children. However, we noted that there was inconclusive evidence for some parameters, suggesting that follow-up with larger samples will be required.

To enable cross-syndrome comparisons, we then applied this same paradigm, using both motion and orientation tasks, to children with dyslexia in a pre-registered study (Manning, Hulks, et al., 2022). In the motion tasks, we found two significant differences. First, children with dyslexia had higher internal noise levels than typically developing children, reflecting reduced precision when estimating local dot directions, and they had higher motion coherence thresholds, in line with previous work (Benassi et al., 2010). However, there were no group differences in sampling, showing that children with dyslexia did not show the same pattern as that found in autism. Meanwhile, in the orientation tasks, the children with dyslexia had higher orientation coherence thresholds than typically developing children (in line with Conlon et al., 2009 and Hansen et al., 2001), but no significant differences in sampling and internal noise. This pattern was again in contrast to the autism data, where we found no evidence of group differences in orientation tasks. This approach has therefore been useful in identifying divergence in autistic and dyslexic children’s perceptual performance. It has also been applied to adult clinical populations, such as those with migraine (O’Hare et al., 2021; Tibber et al., 2014) and schizophrenia (Tibber et al., 2015), enabling broader cross-syndrome insights. In summary, while motion coherence thresholds have been shown to be elevated in a range of conditions, the underlying spatial parameters contributing to performance appear to vary from condition to condition.

## Uncovering temporal dynamics using diffusion modelling and EEG

**Figure 2.**
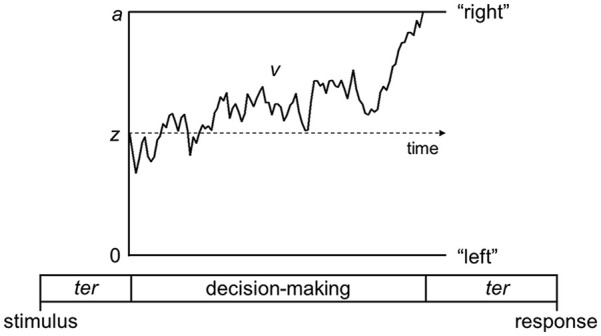
Schematic representation of the decision-making process in the diffusion model for a trial with rightward motion. Source. Figure reproduced from Manning, Hassall, Hunt, Norcia, Wagenmakers, Snowling, et al. (2022). Decision-making process represented as a noisy accumulation of evidence from a starting point, z, towards one of the two decision bounds. In Manning, Hassall, Hunt, Norcia, Wagenmakers, Snowling, et al. (2022) and Manning, Hassall, Hunt, Norcia, Wagenmakers, Evans, and Scerif (2022), participants discriminated between leftward and rightward motion as quickly and accurately as possible, so the decision bounds corresponded to left and right responses. Boundary separation, a, represents the width between the two bounds and reflects response caution. Wider decision boundaries reflect that more evidence is required before making a decision (i.e., more cautious responses). Drift rate, v, reflects the rate of evidence accumulation, which depends on both the individual’s sensitivity to a stimulus and the stimulus strength. Nondecision time, ter, is the time taken for sensory encoding processes prior to the decision-making process and response generation processes after a bound is reached.

The work I have reviewed up to this point has uncovered spatial parameters contributing to performance in perceptual tasks. However, it is also important to recognise the dynamic processes that unfold over time when presented with a visual stimulus and asked to make decisions about it, as these temporal dynamics could also reveal important sources of differences between those with autism and dyslexia. The diffusion model is a popular model of decision-making which decomposes performance in perceptual tasks into distinct processing stages (Ratcliff & McKoon, 2008; White et al., 2010). Unlike the equivalent noise model which models the accuracy of responses, the diffusion model also takes response time distributions into account. The idea is that when making a perceptual decision which requires a binary choice, evidence is accumulated in a noisy fashion until one of two decision boundaries is reached (Figure 2). When discriminating the overall motion direction, these decision boundaries could be for “left” and “right” responses. One important parameter is the drift-rate, which reflects how quickly evidence is accumulated, which varies from person-to-person (reflecting their underlying sensitivity) and across stimulus conditions (reflecting the strength of sensory evidence within a stimulus). Another important parameter is boundary separation, which reflects how far apart the decision boundaries are, and thus how much evidence will be accumulated before making a decision. The boundary separation parameter reflects speed-accuracy tradeoffs: very narrow bounds show a prioritisation of speed over accuracy (relatively more risky decisions), whereas very wide bounds show a prioritisation of accuracy over speed (relatively more cautious decisions). Finally, non-decision time reflects sensory encoding and response generation processes that occur outside of the decision process but contribute to the overall response time.

We applied this model to understand motion coherence performance in typically developing children aged 6 to 12 years and adults, who were asked to discriminate the direction of motion and respond with a button-press as quickly and accurately as possible (Manning et al., 2021). We found that children had lower drift-rates (reduced sensitivity), wider boundary separation (more cautious decisions) and longer non-decision times (more time taken for sensory encoding and/or response generation) than adults (Manning et al., 2021; see also Ratcliff et al., 2012, for similar findings with different decision-making tasks), with the best fitting model allowing age to covary with drift-rate and boundary separation. We also complemented this modelling approach with high-density EEG, which, by virtue of its high temporal resolution, can provide complementary insights into underlying dynamic processes (Kelly & O’Connell, 2013; O’Connell et al., 2012). Using a data-driven, dimension-reduction approach which extracts components that maximise spatiotemporal trial-to-trial reliability (Reliable Components Analysis; Dmochowski & Norcia, 2015), we found two EEG components with distinct topographies and timecourses (Manning et al., 2019). One of these components was a component which was maximal over occipital electrodes and had a negative peak at ~300 ms (like the N2 over occipital electrodes; Niedeggen & Wist, 1998, 1999). The other was a sustained positive component that was maximal over centro-parietal electrodes, which had an amplitude that steadily rose prior to the response, resembling the centro-parietal positivity (Dmochowski & Norcia, 2015; Kelly & O’Connell, 2013). Both of these components showed age-related differences, which, following previous work with adults (Dmochowski & Norcia, 2015; Kelly & O’Connell, 2013; Niedeggen & Wist, 1998, 1999), we attributed to developmental changes in sensory encoding and decision-making processes, respectively. Moreover, we found that the rate at which the amplitude ramped up prior to the response in the centro-parietal component was related to the drift-rate parameter in the diffusion model (Manning et al., 2021). This means that young children accumulate sensory evidence related to motion more slowly than older children and adults, and that this is accompanied by a neural correlate, with shallower ramping up of amplitude in the centro-parietal component.

We next applied this combined modelling and EEG approach to investigate which processing stages might differ in autistic and dyslexic children. Here we presented children with both a coherent motion task and a Gaussian motion task, following the equivalent noise studies reviewed in section “Uncovering spatial parameters using equivalent noise modelling,” which suggested that these tasks might lead to different patterns of performance in autistic children due to their differential demands on noise exclusion processes. We first looked at the EEG data alone and found that neither the group of autistic children nor the group of dyslexic children differed in amplitude from the group of typically developing children in the early, N2-like component over occipital electrodes, following the onset of global motion, for either motion task (Toffoli et al., 2021). This finding aligns with other studies which have showed no evidence of N2 peak amplitude differences in dyslexia (Scheuerpflug et al., 2004; Taroyan et al., 2011), but contrasts a study which showed reduced amplitudes in autistic children (Greimel et al., 2013), which could be due to differences in stimulus parameters and a considerably smaller sample size (*n* = 16 autistic; n = 12 typically developing) compared with our own (n = 29 autistic, n = 57 typically developing). Interestingly, in our study there was some initial evidence that both autistic and dyslexic children’s amplitudes in the occipital component differed from typically developing children at later timepoints, around ~430 ms after stimulus onset, specifically for the motion coherence task (and not the Gaussian motion task), which we tentatively suggested could reflect atypical noise exclusion processes in both autism and dyslexia. Notably, Schulte-Körne et al. (2004) also suggested that differences between dyslexic and typically developing individuals’ evoked responses to motion coherence only emerged at later processing stages (around 300–800 ms).

We then investigated decision-making parameters using the diffusion model in further pre-registered studies with a blind-modelling approach (Manning, Hassall, Hunt, Norcia, Wagenmakers, Evans, & Scerif, 2022; Manning, Hassall, Hunt, Norcia, Wagenmakers, Snowling, et al., 2022). Children with dyslexia showed an overall reduced drift-rate compared with typically developing children, across both tasks (see also O’Brien and Yeatman, 2021, who reported the same for a motion coherence task), showing that they generally accumulated motion evidence more gradually, regardless of the relative noise exclusion demands of the task (Manning, Hassall, Hunt, Norcia, Wagenmakers, Snowling, et al., 2022). This result mirrors the fact that group differences in performance were reported between dyslexic and typically developing children in both motion coherence and Gaussian motion tasks in “Uncovering spatial parameters using equivalent noise modelling.” We also found that dyslexic children had a shallower ramping up of amplitude in the centro-parietal component (as in young typically developing children [Manning et al., 2021]); a result also reported by Stefanac et al. (2021). Using a joint modelling approach, we then showed that this shallower ramping up of amplitude was linked to reduced drift-rate in dyslexic children, thereby linking brain and behaviour (Manning, Hassall, Hunt, Norcia, Wagenmakers, Snowling, et al., 2022). Meanwhile, there was no conclusive evidence for differences in boundary separation and non-decision time between dyslexic and typically developing children. When comparing autistic children to typically developing children, we found no evidence for group differences in any diffusion model parameter. This finding was in contrast to our hypotheses based on previous work which has showed increased boundary separation (i.e., more cautious responses) in autistic individuals compared with neurotypical individuals (Iuculano et al., 2020; Pirrone et al., 2017, 2020). While we found a mean group difference in boundary separation in the expected direction, the groups were highly overlapping, with much between-participants variability, so that we did not find clear evidence in terms of Bayes factors (BF > 3). Moreover, the ramping up of amplitude in the centro-parietal component did not consistently relate to evidence accumulation in autistic children (Manning, Hassall, Hunt, Norcia, Wagenmakers, Evans, & Scerif, 2022), as it did in typically developing children (Manning et al., 2021). The fact that no group differences were found in this study between autistic and typically developing participants, despite previous reports of group differences in motion processing tasks, could be due to the stimulus difficulty levels chosen (see discussion in Manning, Hassall, Hunt, Norcia, Wagenmakers, Evans, & Scerif, 2022). The lack of clear evidence for increased boundary separation in autistic participants relative to typically developing participants (unlike Iuculano et al., 2020; Pirrone et al., 2017, 2020), could be due to our chosen modelling technique which accounts for uncertainty in individual-level parameters when making inferences. Another possibility is that the explicit instructions we gave to participants asking them to respond both quickly and accurately, in contrast to some previous studies, affected their decision-making strategies. However, from our set of cross-syndrome studies using the same paradigm and analysis approach, it appears that there is divergence in autistic and dyslexic children’s perceptual decision-making, whereby reduced drift-rates and the associated shallower build-up of activity over centro-parietal electrodes are specific to dyslexia.

## Overall conclusion

Summarising the results of these approaches together, we have identified age-related differences in both the spatial parameters and temporal dynamics involved in motion processing tasks, which can help to further understand the protracted development of motion processing abilities through childhood. Specifically, compared with older children and adults, younger children are less able to average motion signals over space, and they extract sensory evidence from motion displays more gradually, while also being more cautious in their decision-making style. They also show differences in their neural responses which appear to reflect early sensory encoding and later decisional processing. These results are based on cross-sectional studies, so longitudinal studies will be needed to investigate further how these parameters change over developmental time.

Our studies with autistic and dyslexic children have identified both areas of convergence and divergence in processing of visual motion and orientation information in these conditions. Autistic children showed an increased ability to average motion information compared with typically developing children using the equivalent noise approach, but showed no evidence of group differences in equivalent noise orientation tasks or in diffusion model parameters. However, children with dyslexia showed increased internal noise for motion processing, and elevated motion and orientation coherence thresholds compared with typically developing children in the equivalent noise approach. Then in the diffusion modelling approach, children with dyslexia showed a reduced accumulation of sensory evidence in both motion tasks, and a shallower build-up of amplitude in the centro-parietal EEG component. At this point, it is worth noting that, across both approaches, the children with dyslexia appeared to show greater difficulties with motion processing than autistic children, overall, although we note that the group differences are still quite subtle and that there is much overlap between the groups. There were also areas of convergence between autistic and dyslexic children, including that their early EEG responses to motion appeared similar to typically developing children, with differences only appearing at later processing stages (which may reflect reduced noise exclusion across both conditions; Toffoli et al., 2021).

These results have implications for theories. We did not find evidence in support of the weak central coherence account of autism (Frith & Happé, 1994; Happé & Frith, 2006), because autistic children did not show reductions in integrative abilities in either motion or orientation processing tasks relative to typically developing children. Instead, we showed an area of *enhanced* integrative ability for autistic children, in a motion processing task, when there was no requirement for noise exclusion. We also found no clear evidence for group differences in internal noise in autistic children (Davis & Plaisted-Grant, 2015; Simmons et al., 2009), although other paradigms may reveal group differences in internal noise (Park et al., 2017; and see Vilidaite et al., 2017 and Orchard et al., 2022, showing increasing levels of internal noise as a function of autistic traits). The pattern of performance we found for children with dyslexia in motion tasks is partially consistent with the magnocellular theory (Stein, 2019; Stein & Walsh, 1997), in that we found elevated motion coherence thresholds and elevated internal noise in motion tasks. However, our EEG and diffusion modelling suggests that later, decision-making processes are affected, rather than early visual encoding which would be attributed to the magnocellular system. We also reported elevated orientation coherence thresholds in children with dyslexia, and we did not test whether reduced evidence accumulation might generalise to non-motion tasks, so future work is needed to test the domain-generality here. The related dorsal-stream vulnerability account has been proposed to explain elevated motion coherence thresholds (relative to orientation coherence thresholds) in a range of conditions including autism and dyslexia (Atkinson, 2017; Braddick et al., 2003). The findings reviewed here suggest this account needs refining as we report an area of enhanced motion processing ability in autism (see also Foss-Feig et al., 2013), and because we also find elevated orientation coherence thresholds in dyslexic children. Moreover, the fact that we find areas of divergence in what underlies autistic and dyslexic children’s performance suggests a need to move beyond accounts that explain commonalities across conditions. Future work would then be needed to establish whether any of the condition-specific patterns of spatial parameters and temporal dynamics play a causal role in the development of these conditions, or instead, to explain how these condition-specific patterns emerge through development.

## Future directions

The work reviewed here considered the equivalent noise model and the diffusion model separately, but it would be informative to develop a framework by which the two modelling approaches could be combined. This would help us understand, for example, how the increased internal noise identified in dyslexic children in equivalent noise modelling relates to reduced drift-rate in the diffusion model. Future work is also needed to identify the conditions under which group differences emerge—for example, we found enhanced performance in autistic children in a Gaussian motion task when using our equivalent noise approach (Manning et al., 2015), but not when using two fixed difficulty levels in the diffusion modelling approach (Manning, Hassall, Hunt, Norcia, Wagenmakers, Evans, & Scerif, 2022). We also note that previous studies have reported differences in diffusion modelling parameters in autistic participants (Iuculano et al., 2020; Karalunas et al., 2018; Kirchner et al., 2012; Pirrone et al., 2017, 2020), and this discrepancy could be due to choice of task, task instructions about speed/accuracy emphasis, and/or analytical choices. These are questions for future work, including ongoing studies in our lab.

It would also be interesting to investigate how these parameters manifest in autistic and dyslexic adults, as differences in developmental maturation may contribute to individual differences in adults. To my knowledge, there is no published work applying the equivalent noise paradigm described here to autistic and dyslexic adults. There is work which has applied diffusion modelling to autistic adults and reported group differences in model parameters (e.g., Pirrone et al., 2017), although no studies which have compared across conditions.

More notable than the presence or absence of group differences is the considerable individual differences between participants that we found across our studies. Even where there was evidence of group differences, these tended to be relatively small, with much overlap between groups. Future work is needed to understand this variability. It is also important to recognise that there are no clear-cut distinctions between different developmental conditions, with conditions commonly co-occurring (Gillberg, 2010). Therefore, future work would benefit from taking a transdiagnostic approach (Astle et al., 2022), by looking at how continuous dimensions related to autism, dyslexia and other aspects of neurodiversity affect the spatial and temporal parameters linked to visual perception. In our autism work, we found preliminary evidence that continuous attention deficit hyperactivity disorder (ADHD) traits are related to drift-rate in the diffusion model (Manning, Hassall, Hunt, Norcia, Wagenmakers, Evans, & Scerif, 2022), showing that this is another dimension worth studying across the population. Such a transdiagnostic approach may help to understand individual differences more generally (Manning & Scerif, 2023).
